# Complications during Veno-Venous Extracorporeal Membrane Oxygenation in COVID-19 and Non-COVID-19 Patients with Acute Respiratory Distress Syndrome

**DOI:** 10.3390/jcm13102871

**Published:** 2024-05-13

**Authors:** Andrea Bruni, Caterina Battaglia, Vincenzo Bosco, Corrado Pelaia, Giuseppe Neri, Eugenio Biamonte, Francesco Manti, Annachiara Mollace, Annalisa Boscolo, Michele Morelli, Paolo Navalesi, Domenico Laganà, Eugenio Garofalo, Federico Longhini, IMAGE Group

**Affiliations:** 1 Department of Medical and Surgical Sciences, “Magna Graecia” University, 88100 Catanzaro, Italy; andreabruni@unicz.it (A.B.); vincenzo.bosco@unicz.it (V.B.); pelaia.corrado@gmail.com (C.P.); giuseppeneri91@gmail.com (G.N.); domenico.lagana@unicz.it (D.L.); longhini.federico@gmail.com (F.L.); 2 Radiodiagnostic Institute, Dulbecco Hospital, 88100 Catanzaro, Italy; caterina.battaglia@unicz.it (C.B.); mantifra@unicz.it (F.M.); annachiaramollace@gmail.com (A.M.); 3 Institute of Anesthesia and Intensive Care, Dulbecco Hospital, 88100 Catanzaro, Italy; egbiam@yahoo.it; 4 Department of Medicine (DIMED), University of Padua, 35131 Padua, Italy; annalisa.boscolo@gmail.com (A.B.); paolo.navalesi@unipd.it (P.N.); 5 Institute of Anesthesia and Intensive Care, Padua University Hospital, 35122 Padova, Italy; 6 Thoracic Surgery and Lung Transplant Unit, Department of Cardiac, Thoracic, Vascular Sciences, and Public Health, University of Padua, 35122 Padova, Italy; 7 Department of Obstetrics and Gynecology, “Annunziata” Hospital, 87100 Cosenza, Italy; morellimichele122@gmail.com

**Keywords:** extracorporeal membrane oxygenation, acute respiratory distress syndrome, complication, COVID-19, pneumothorax, barotrauma, mortality

## Abstract

**Background:** Acute respiratory distress syndrome (ARDS) presents a significant challenge in critical care settings, characterized by compromised gas exchange, necessitating in the most severe cases interventions such as veno-venous extracorporeal membrane oxygenation (vv-ECMO) when conventional therapies fail. Critically ill ARDS patients on vv-ECMO may experience several complications. Limited data exist comparing complication rates between COVID-19 and non-COVID-19 ARDS patients undergoing vv-ECMO. This retrospective observational study aimed to assess and compare complications in these patient cohorts. **Methods:** We retrospectively analyzed the medical records of all patients receiving vv-ECMO for ARDS between March 2020 and March 2022. We recorded the baseline characteristics, the disease course and complication (barotrauma, bleeding, thrombosis) before and after ECMO cannulation, and clinical outcomes (mechanical ventilation and ECMO duration, intensive care unit, and hospital lengths of stay and mortalities). Data were compared between COVID-19 and non-COVID-19 patients. In addition, we compared survived and deceased patients. **Results:** Sixty-four patients were included. COVID-19 patients (n = 25) showed higher rates of pneumothorax (28% vs. 8%, *p* = 0.039) with subcutaneous emphysema (24% vs. 5%, *p* = 0.048) and longer non-invasive ventilation duration before vv-ECMO cannulation (2 [1; 4] vs. 0 [0; 1] days, *p* = <0.001), compared to non-COVID-19 patients (n = 39). However, complication rates and clinical outcomes post-vv-ECMO were similar between groups. Survival analysis revealed no significant differences in pre-vv-ECMO complications, but non-surviving patients had a trend toward higher complication rates and more pleural effusions post-vv-ECMO. **Conclusions:** COVID-19 patients on vv-ECMO exhibit higher pneumothorax rates with subcutaneous emphysema pre-cannulation; post-cannulation complications are comparable to non-COVID-19 patients.

## 1. Introduction

Acute respiratory failure (ARF) is a pathological condition characterized by altered gas exchange secondary to impaired function of the respiratory muscle pump and/or to dysfunction of the lung [1,2]. Its incidence is reported to be around 77–88 cases per 100,000 inhabitants, with higher values in middle-aged subjects and even higher in elderly subjects [2].

In the most severe cases of ARF and acute respiratory distress syndrome (ARDS) [1,2], veno-venous extracorporeal membrane oxygenation (vv-ECMO) can be implemented if the patient is refractory to conventional treatments, the etiology is potentially reversible, and there are no formal contraindications for the initiation of this support [3]. Cannulation for vv-ECMO entails extracting blood from the patient’s venous system (i.e., femoral vein) using a large drainage cannula. After being forced by a centrifugal pump through an oxygenator for gas exchange, the oxygenated blood is reintroduced back into the venous system (i.e., contralateral femoral vein or jugular vein) using a return cannula [3,4].

Following the insertion of ECMO cannulas, a chest and abdomen X-ray is typically required to verify their accurate placement, assess the spacing between them, and identify any possible complications like pneumothorax or hemothorax [3,5]. Imaging for cannula placement may also involve echocardiographic (TEE) guidance depending on the cannula [3]. As a first-level examination and follow-up, chest X-ray is then commonly repeated every 2–3 days, together with other imaging techniques [6,7,8,9]. 

Patients with severe ARDS and receiving vv-ECMO may be affected by complications associated with mechanical ventilation (barotrauma) and/or vv-ECMO treatment (including thromboembolisms, hemorrhage) [10,11,12]. This particularly holds true in patients affected by SARS-CoV-2 infection [13,14]. Nevertheless, there are limited data available regarding the occurrence of complications in patients with SARS-CoV-2-related ARDS compared to those with non-SARS-CoV-2-related ARDS undergoing ECMO therapy, within the same period of treatment. We have, therefore, conducted this retrospective observational study to compare the occurrence of complications in patients undergoing vv-ECMO because of ARDS related or not to SARS-CoV-2.

## 2. Methodology

This retrospective study was conducted in the period between March 2020 and March 2022 at the Intensive Care Unit of “Renato Dulbecco” University Hospital in Catanzaro (Italy). The study was approved by the local Ethical Committee of the Calabria Region (approval n. 7/2023, on 27 September 2023). Written informed consent was waived due to the retrospective study design.

### 2.1. Patients

All adult (i.e., age > 18 years/old) patients receiving vv-ECMO for ARDS were included in the study. 

We excluded patients with chronic respiratory failure conditions such as cystic fibrosis who were started on vv-ECMO explicitly for bridging to lung transplant, as well as patients undergoing vv-ECMO for refractory hypercapnia due to status asthmaticus or exacerbations of chronic obstructive pulmonary disease. This exclusion was made because complications and outcomes of these patients differ from those with hypoxemic respiratory failure who receive vv-ECMO [15,16,17].

Indications to vv-ECMO are those outlined in the current guidelines from the Extracorporeal Life Support Organization (ELSO) [3,18]. In particular, we considered candidates for vv-ECMO, all severe ARDS patients with refractory hypoxemia (as defined by an arterial partial pressure to inspired fraction of oxygen ratio [PaO_2_/FiO_2_] < 80 mmHg), or severe hypercapnic respiratory failure (as defined by an arterial pH < 7.25 with an arterial partial pressure of carbon dioxide [PaCO_2_] ≥ 60 mmHg), after optimal conventional management (including, in the absence of contraindications, a trial of prone positioning) [3,18]. If one or more of the following criteria was present, vv-ECMO was not implemented: (1) central nervous system hemorrhage or severe injury, determining an irreversible and incapacitating pathology; (2) systemic bleeding; (3) contraindications to anticoagulation; (4) immunosuppression; or 5) mechanical ventilation for more than 7 days with a plateau pressure > 30 cmH_2_O and FiO_2_ > 90% [3,18].

After cannulation, a chest and abdomen X-ray was acquired to verify their placement and to identify possible complications like pneumothorax or hemothorax [3,5]. Ventilator settings were subsequently modified to reflect resting ventilation parameters. Specifically, patients were ventilated using volume-controlled mode, with a tidal volume ranging from 3 to 6 mL per kg of ideal body weight, while ensuring a driving pressure < 13 cmH_2_O and maintaining a respiratory rate between 4 and 6 breaths per minute. If oxygenation and arterial partial pressure of carbon dioxide (PaCO_2_) targets were not achieved, adjustments were made to the ECMO circuit rather than increasing ventilator settings [3,5]. PEEP was determined with electrical impedance tomography and remained unchanged after the ECMO cannulation [6]. Ventilator settings, including driving pressure and PEEP, were verified every 8 h. Anticoagulation with unfractionated heparin, fluid management, and procedure of weaning from vv-ECMO were conducted as per ELSO guidelines [3,5,19]. 

### 2.2. Data Collection and Analysis

All patients meeting the inclusion criteria were categorized upon hospital admission as either non-COVID-19 or COVID-19 based on the presence of SARS-CoV-2 detected via reverse transcriptase-polymerase chain reaction testing of nasopharyngeal swabs [20].

In both study groups, we collected the following variables: age, gender, body mass index, pre-existing comorbidities, primary etiological diagnosis of the ARDS, PaO_2_/FiO_2_ ratio, and treatments received before vv-ECMO. We also recorded the following complications occurred before or after vv-ECMO cannulation, as assessed through chest X-ray, CT scan, blood tests, and/or ultrasonography: pneumothorax, pneumomediastinum, subcutaneous emphysema, pleural effusion, hemothorax, acute myocardial infarction, abdominal effusion, abdominal bleeding, ischemic or hemorrhagic brain injury, and thromboembolic complications (including limb ischemia and deep vein thrombosis).

Finally, we collected the duration of vv-ECMO and (invasive and non-invasive) mechanical ventilation, the rate of tracheostomy, the ICU, and hospital lengths of stay and mortalities. Total time spent under mechanical ventilation has been computed as the sum of the days spent under invasive plus non-invasive ventilation.

### 2.3. Statistical Analysis

The Anderson–Darling test was applied to investigate whether data were normally distributed. Data were expressed as mean ± standard deviation (SD) if normally distributed, otherwise as median values with interquartile range (IQR). The occurrence of complications was described in absolute and relative frequencies. All data have been reported for the overall population and in COVID-19 and non-COVID-19 groups, separately. All data have been also analyzed as per hospital mortality. Given the small sample of patients, we preferred to conduct a non-parametric statistical analysis to be more conservative in the results [21]. Categorical variables have been compared between COVID-19 and non-COVID-19 groups with the Fisher’s exact test; the odds ratio [95% Confidence of Interval] has been also reported. Continuous variables have been compared between groups through the Mann–Whitney U-test. Since time-to-event exposure was different between survivors and non-survivors, for all complications, we computed the exposure standardized incidence rate per 1000 days of ICU Length of stay. Incidence rates were compared between survived and died patients through the incidence rate ratio; incidence rate ratio [95% CI] has been also reported. A *p*-value less than 0.05 was statistically significant. Statistical analysis was performed using Prism version 9.5.1 (GraphPad Software Inc., San Diego, CA, USA).

## 3. Results

Sixty-eight critically ill patients receiving vv-ECMO were identified within the study period. Of note, four patients were excluded (one on vv-ECMO explicitly for bridging to lung transplant, three undergoing vv-ECMO for refractory hypercapnia due to status asthmaticus). Therefore, we included 64 patients. Thirty-nine patients (61%) experienced hypoxemic ARF unrelated to a SARS-CoV-2 infection (referred to as the “non-COVID-19” subgroup), while 25 patients (39%) tested positive for SARS-CoV-2 (referred to as the “COVID-19” subgroup). The baseline characteristics of both the entire patient population and the two subgroups are presented in Table 1. It is noteworthy that among the non-COVID-19 patients, two individuals, and among the COVID-19 group, six individuals had additional (bacterial, viral, or fungal) infections concurrent with the primary infection upon hospital admission. Of note, none had malignancies, immunosuppressive therapies, or pregnancy before ARDS onset.

### 3.1. Complications in COVID-19 and Non-COVID-19 Patients

Table 2 shows the results concerning the clinical conditions, complications, and some clinical outcomes before the vv-ECMO cannulation. It needs to be highlighted that non-COVID-19 patients were characterized by a higher need for vasopressors, as compared to COVID-19 patients (87% vs. 48%, *p* = 0.001). Thirty-five (55%) patients had one or more complications before vv-ECMO cannulation, 17 among non-COVID-19 patients and 18 among COVID-19 ones (44% vs. 72%, *p* = 0.039). Among non-COVID-19 patients, seven patients experienced a single complication, seven patients had two concurrent complications, and three patients exhibited three complications simultaneously. Conversely, among COVID-19 patients, eight patients had one single complication, four patients had two complications, and six patients had three or more concurrent complications. In addition, as opposed to non-COVID-19 patients, COVID-19 patients had a higher rate of pneumothorax (28% vs. 8%, *p* = 0.039) with concomitant subcutaneous emphysema (5% vs. 24%, *p* = 0.048). Finally, as opposed to non-COVID-19 patients, patients in the COVID-19 group had a higher rate of NIV use (41% vs. 80%, *p* = 0.004) for a longer time (*p* < 0.001) and a higher hospital LOS (*p* = 0.019) (see Table 2).

Table 3 reports the recorded complication newly occurred after vv-ECMO cannulation in the overall population, and in non-COVID-19 and COVID-19 patients, separately. In our study population, the number of patients with one or more complications after vv-ECMO cannulation, and the type of occurred complications were similar between groups. Of note, we did not record in any patient abdominal effusion or bleeding, or cerebral or limb ischemia. Of note, among non-COVID-19 patients, two experienced a single complication, while 10 patients exhibited three or more complications simultaneously. Conversely, among COVID-19 patients, four patients had one single complication, one patient had two complications, and six patients had three or more concurrent complications.

Finally, assessed clinical outcomes were similar between subgroups of patients (see Table 4).

### 3.2. Complications in Survived and Non-Survived Patients

As shown in Table 5, there were similarities in clinical conditions, complications, and certain clinical outcomes prior to vv-ECMO cannulation among both survived and non-survived patients. The rates of patients with one or more complications were similar between survivors and non-survivors (68% vs. 83%, *p* = 0.252). Notably, there was a tendency towards significance regarding the length of hospital stay, which was higher in non-survived patients (*p* = 0.051).

Table 6 presents the exposure-standardized complications per 1000 days of ICU admission that occurred after vv-ECMO cannulation in both survived and non-survived patients. Non-surviving patients exhibited a higher incidence rate of pneumothorax, subcutaneous emphysema, pulmonary embolism, pleural effusion, acute myocardial infarction, hemorrhagic brain injury, and deep vein thrombosis compared to survivors.

Table 7 displays the clinical outcomes observed between surviving and deceased patients. There were notable differences in the majority of clinical outcomes between the two groups. It is worth mentioning that 21 patients died within the first week following vv-ECMO cannulation, while the remaining two patients passed away after 14 and 16 days due to septic shock. All deceased patients died before vv-ECMO weaning and decannulation.

## 4. Discussion

In this retrospective observational study, we found that COVID-19 patients have a higher rate of pneumothorax and related subcutaneous emphysema before ECMO cannulation compared to non-COVID-19 patients. In addition, a higher percentage of COVID-19 patients received NIV for a longer time before vv-ECMO cannulation. Conversely, complications after vv-ECMO cannulation were similarly represented in the two subgroups of patients. 

In COVID-19 patients, there is a disproportionate association with barotrauma (pneumothorax, pneumomediastinum, and subcutaneous emphysema), compared to traditional ARDS cases [22]. Indeed, up to 16% of COVID-19 patients with ARDS and receiving iMV may develop barotrauma, with a mortality rate > 60% [23]. In addition, COVID-19 patients receiving both NIV or iMV have a higher incidence of barotrauma, as compared to those receiving standard or high-flow nasal cannula oxygen therapy [14]. Those differences may be explained by several conditions. First, patients developing barotrauma are tachypneic [24,25,26]; this may be associated also with a high respiratory effort [24,27,28], with increased risk of generating excessive transpulmonary pressure and patient-self-inflicted lung injury (P-SILI) [7,29]. Second, the involvement of the central nervous system by the viral infection alters the respiratory drive, leading also to increased transpulmonary pressure, global and regional strain (resulting in barotrauma), pronounced inflammatory profile, and P-SILI [14,24,30]. Third, the lungs of COVID-19 patients with ARDS are considered frail. In the CT scan of patients developing barotrauma, the Macklin effect can be very commonly identified [31]. The Macklin effect appears on thoracic CT as linear collections of air contiguous to the bronchovascular sheaths [32]. The Macklin effect has been recognized as a consistent radiological predictor of barotrauma development in COVID-19 ARDS patients, 8 to 12 days before clinically overt barotrauma [31,33,34]. For this reason, it has been proposed and demonstrated safe to manage COVID-19 patients with Macklin effect at the CT scan with awake vv-ECMO. This strategy is grounded in the rationale that vv-ECMO enables patients to circumvent numerous side effects associated with prolonged sedation and paralysis. It also diminishes the risk of secondary infections linked to prolonged iMV and the need for additional invasive procedures, such as tracheostomy. Additionally, patients on awake vv-ECMO can engage in early active physiotherapy, offering potential long-term advantages and a shorter recovery time in terms of functional capacity and neuropsychological recuperation [35,36]. These aspects may explain our findings on the increased number of patients with barotrauma in the COVID-19 subgroup, as opposed to non-COVID-19 patients. It should be mentioned that the duration of NIV and the hospital LOS before ECMO cannulation were longer in the COVID-19 subgroup of patients. This may have further increased the percentage of patients with barotrauma since the effects exerted by increased respiratory effort and transpulmonary pressure during NIV were sustained for a longer time [24]. Indeed, NIV should be approached cautiously in cases of hypoxemic de novo ARF [37], as the primary concern is the potential for delaying necessary intubation, which can result in a worsened patient outcome [38,39]. Furthermore, it is well demonstrated that delay in intubation and NIV prolongation in COVID-19 patients may drastically affect clinical outcomes [40,41,42,43]. However, during the COVID-19 pandemic, the critical care surge capacity was exceeded by the request for mechanical ventilation for patients [44]. This emergency led physicians to prolong the time under NIV, extending its indications and developing new approaches to patient care [45,46,47,48,49,50].

It is interesting, however, that we could not find differences among complications between ARDS patients with or without SARS-CoV-2 infection after vv-ECMO cannulation. This may be explained by the standardized management strategy between subgroups of patients for mechanical ventilation and for anticoagulants [3]. Although the rates of complications are similar, pulmonary embolism was three times higher in the COVID-19 group (though not reaching statistical differences). It is well known that critically ill COVID-19 patients suffer from a hypercoagulative status [51] that might be resistant to heparin, therefore, requiring alternative anticoagulant therapy [52,53].

It is noteworthy that we observed no differences in clinical outcomes between COVID-19 and non-COVID-19 patients. Our findings should, however, be considered with caution due to the small sample of patients (see below). To date, there have been few studies examining and contrasting the outcomes of COVID-19 patients undergoing vv-ECMO with those of non-COVID-19 patients receiving the same treatment. Dave et al. compared 35 COVID-19 and 54 non-COVID-19 patients, with a quite similar sample of patients [54]. The non-COVID-19 population consisted of patients with pneumonia due to influenza virus. Interestingly they reported a higher incidence of pneumothorax and mortality rate in COVID-19 patients [54]. It should be noted that they reported pneumothorax in 60% of COVID-19 patients, a rate similar to that in our population. In fact, we identified pneumothorax in seven patients before ECMO cannulation plus eight patients after ECMO cannulation (15/25 patients, 60%). Mortality was also similar between the two studies [54].

Garfield et al. also compared 53 patients with COVID-19 ARDS admitted for vv-ECMO with a historical cohort of patients with non-COVID-19 viral pneumonia [55]. Compared to our findings, they reported a lower incidence of pneumothorax and mortality and a higher rate of COVID-19 patients complicated by pulmonary embolism [55]. Another recent study by Kim et al. compared the clinical outcomes of 21 COVID-19 patients with 24 non-COVID-19 (varying etiologies) patients [56]. The study population was very similar to ours as per age, etiology, severity of the ARF, and disease course before vv-ECMO cannulation [56]. Of note, our clinical outcomes are partially in keeping with those reported by other larger trials [57,58]. In fact, in a large and multicenter study from the ELSO registry, Barbaro et al. showed mortality to be higher in COVID-19 patients, being around 40% (close to our findings) [58].

We also assessed the differences between survivors and non-survivors. It is interesting that deceased patients had a higher rate of pleural effusion compared to survivors. In critically ill patients with ARDS, pleural effusion is frequently related to fluid overload, hyperoncotic states, or heart failure [59]. It is well known that positive fluid balance is associated with a higher mortality rate [60,61]. Although initial fluid resuscitation is recommended by the sepsis and septic shock guidelines [62], the need for more fluid could be due to increased vascular permeability [63]. Vascular permeability is a selective mechanism that maintains the exchange between vessels, tissues, and organs, which is compromised in case of dysregulated inflammatory response as during septic shock or ARDS [64]. A more pronounced inflammatory status is associated with severe organ failures and mortality [65]. Indeed, employing extracorporeal hemoadsorption to remove cytokines within the initial 87 h of ICU admission has demonstrated efficacy in decreasing the duration of ventilation and vv-ECMO support, as well as reducing ICU LOS among COVID-19 patients [66].

Before drawing our conclusions, some limitations must be discussed. First, this study has a retrospective observational design, that may be affected by selection bias, and the findings may not be representative of a broader population; in addition, there could be several confounding variables preventing the possibility to investigate casualties [67]. Additionally, the retrospective design also limited our ability to assess certain crucial data across the entire population, such as fluid balance. Notably, strategies like conservative fluid management or deresuscitation (involving active removal of fluid through diuretics or CRRT) are recognized for their potential to enhance clinical outcomes, including reducing the duration of ventilatory support and ICU length of stay [68,69]. However, it is important to note that in our daily clinical practice, we strive to achieve negative fluid balances in ARDS patients. Second, the study has been conducted in a single center, limiting the generalizability of our findings to other populations or settings. However, as discussed above, most of our findings confirm data already presented in the literature. Last, the sample can be considered small. Indeed, other published studies have included much larger populations of patients [57,58]. However, a few small studies have reported a “head-to-head” comparison between COVID-19 and non-COVID-19 patients undergoing vv-ECMO with a similarly small sample of patients [54,55,56]. Being aware of the small, included sample, we conducted a non-parametric statistical analysis to be more conservative in our findings [21].

## 5. Conclusions

Compared to non-COVID-19 patients, COVID-19 patients receiving vv-ECMO are characterized by an increased rate of pneumothorax with associated subcutaneous emphysema before ECMO cannulation. Complications occurring after vv-ECMO cannulation were similar between subgroups of patients. While our findings align with those of other reports, it is imperative that they undergo further scrutiny in larger multicenter trials.

## Figures and Tables

**Table 1:** Baseline characteristics of study patients.

	Overall (n = 64)	Non-COVID-19 (n = 39)	COVID-19 (n = 25)	OR [95% CI]	*p*-Value
Age (year)	57 [44; 64]	58 [44; 65]	54 [42; 62]		0.601
Female sex—n (%)	16 (25%)	11 (28%)	5 (20%)	1.57 [0.47–4.73]	0.561
BMI (kg/m^2^)	27 [23; 31]	27 [23; 33]	25 [23; 30]		0.308
Current smoker—n (%)	27 (42%)	15 (38%)	12 (48%)	0.68 [0.26–1.79]	0.605
SOFA	11 [11; 12]	11 [11; 12]	12 [11; 12]		0.424
PaO_2_/FiO_2_ (mmHg)	98 [77; 110]	102 [78; 110]	80 [72; 110]		0.276
Etiology of ARF—n (%)					
Bacterial	36 (56%)	33 (85%)	3 (12%)		<0.001
Viral	31 (48%)	6 (15%)	25 (100%)		
Fungal	5 (8%)	2 (5%)	3 (8%)		
Comorbidities—n (%)					
Arterial hypertension	28 (44%)	14 (36%)	14 (56%)	0.44 [0.16–1.28]	0.130
Diabetes	22 (34%)	14 (36%)	8 (32%)	1.19 [0.44–3.43]	0.794
Cardiovascular disease	27 (42%)	15 (38%)	12 (48%)	0.68 [0.26–1.79]	0.681
Chronic renal failure	3 (5%)	1 (3%)	2 (8%)	0.30 [0.02–2.77]	0.555
Peripheral vascular disease	2 (3%)	1 (3%)	1 (4%)	0.63 [0.03–12.46]	0.999
Cerebrovascular disease	4 (6%)	3 (8%)	1 (4%)	2.00 [0.28–26.94]	0.999
Liver disease	3 (5%)	1 (3%)	2 (8%)	0.30 [0.02–2.77]	0.555

BMI, body mass index; SOFA, sequential organ failure assessment; PaO_2_/FiO_2_ ratio between the arterial partial pressure and inspired fraction of oxygen; ARF, acute respiratory failure.

**Table 2:** Clinical condition before ECMO cannulation.

	Overall (n = 64)	Non-COVID-19 (n = 39)	COVID-19 (n = 25)	OR [95% CI]	*p*-Value
Vasoactive drugs—n (%)	55 (86%)	34 (87%)	12 (48%)	7.37 [2.18–22.85]	0.001
Pulmonary Vasodilators—n (%)	15 (23%)	8 (21%)	7 (28%)	0.66 [0.23–2.07]	0.553
NMBA—n (%)	60 (94%)	35 (90%)	25 (100%)	0.00 [0.00–1.60]	0.150
Corticosteroids—n (%)	31 (48%)	16 (41%)	15 (60%)	0.46 [0.17–1.34]	0.200
Prone Position—n (%)	42 (66%)	24 (62%)	18 (72%)	0.62 [0.22–1.72]	0.622
Pneumothorax—n (%)	10 (16%)	3 (8%)	7 (28%)	0.21 [0.05–0.97]	0.039
Pneumomediastinum—n (%)	7 (11%)	2 (5%)	5 (20%)	0.22 [0.04–1.21]	0.100
Subcutaneous emphysema—n (%)	8 (12%)	2 (5%)	6 (24%)	0.17 [0.03–0.81]	0.048
Pleural effusion—n (%)	22 (34%)	12 (31%)	10 (40%)	0.67 [0.24–1.87]	0.590
Pulmonary embolism—n (%)	5 (8%)	3 (8%)	2 (8%)	0.95 [0.18–7.72]	0.999
Limb ischemia—n (%)	3 (5%)	2 (5%)	1 (4%)	1.26 [0.14–19.00]	0.999
Deep vein thrombosis—n (%)	11 (17%)	6 (15%)	5 (20%)	0.73 [0.21–2.53]	0.738
NIV—n (%)	36 (56%)	16 (41%)	20 (80%)	0.17 [0.06–0.59]	0.004
NIV (days)	1 [0; 2]	0 [0; 1]	2 [1; 4]		<0.001
iMV—n (%)	64 (100%)	39 (100%)	25 (100%)	n.a.	0.999
iMV (days)	2 [1; 3]	2 [1;3]	2 [1;3]		0.581
ICU LOS (days)	2 [1; 4]	2 [1; 4]	2 [1; 4]		0.587
Hospital LOS (days)	4 [2; 5]	2 [1; 5]	5 [2; 7]		0.019

ECMO, extracorporeal membrane oxygenation; NMBA, neuromuscular blocking agents; NIV, non-invasive ventilation; iMV, invasive mechanical ventilation; ICU, intensive care unit; LOS, length of stay; n.a., not applicable.

**Table 3:** Complications newly occurred after ECMO cannulation.

	Overall	Non-COVID-19	COVID-19	OR [95% CI]	*p*-Value
Patients with new complications—n (%)	21/64 (33%)	12/39 (31%)	9/25 (36%)	0.79 [0.28–2.34]	0.786
Pneumothorax—n (%)	17/54 (31%)	9/36 (25%)	8/17 (44%)	0.41 [0.12–1.31]	0.208
Pneumomediastinum—n (%)	10/57 (18%)	5/37 (14%)	5/20 (25%)	0.47 [0.12–1.81]	0.297
Subcutaneous emphysema—n (%)	11/56 (20%)	6/37 (16%)	5/19 (26%)	0.54 [0.16–2.00]	0.481
Pulmonary embolism—n (%)	3/59 (5%)	1/36 (3%)	2/23 (9%)	0.30 [0.02–2.76]	0.554
Pleural effusion—n (%)	10/42(24%)	7/27 (26%)	3/15 (20%)	1.40 [0.35–5.68]	0.999
Hemothorax—n (%)	3/64 (5%)	2/39 (5%)	1/25 (4%)	1.30 [0.14–19.50]	0.999
Acute myocardial infarction—n (%)	2/64 (3%)	1/39 (3%)	1/25 (4%)	0.63 [0.03–12.46]	0.999
Hemorrhagic brain injury—n (%)	1/64 (2%)	1/39 (3%)	0/25 (0%)	n.a.	0.999
Deep vein thrombosis—n (%)	4/53 (8%)	2/33 (6%)	2/20 (10%)	0.58 [0.08–3.99]	0.581

ECMO, extracorporeal membrane oxygenation; n.a., not applicable.

**Table 4:** Clinical outcomes.

	Overall (n = 64)	Non-COVID-19 (n = 39)	COVID-19 (n = 25)	OR [95% CI]	*p*-Value
Prone position during ECMO—n (%)	32 (50%)	19 (49%)	13 (52%)	0.88 [0.34–2.54]	0.999
ECMO duration (days)	8 [5; 12]	8 [5; 10]	10 [5; 13]		0.618
NIV after ECMO cannulation—n (%)	30 (47%)	21 (53%)	9 (47%)	2.07 [0.72–5.91]	0.204
NIV after ECMO cannulation (days)	3 [3; 4]	3 [3; 4]	4 [3; 4]		0.495
iMV after ECMO cannulation (days)	14 [5; 16]	12 [5; 16]	14 [5; 16]		0.894
Total time spent under ventilatory support (days)	19 [11; 21]	19 [7; 21]	19 [11; 23]		0.428
Tracheostomy—n (%)	13 (20%)	7 (18%)	6 (24%)	0.69 [0.21–2.44]	0.751
CRRT after ECMO cannulation—n (%)	26 (41%)	16 (41%)	10 (40%)	1.04 [0.38–2.82]	0.999
Total ICU LOS (days)	23 [9; 30]	24 [9; 30]	22 [9; 30]		0.787
Total hospital LOS (days)	32 [11; 41]	34 [12; 41]	30 [11; 41]		0.669
ICU mortality	23 (36%)	12 (31%)	11 (44%)	0.57 [0.21–1.53]	0.300
Hospital mortality	23 (36%)	12 (31%)	11 (44%)	0.57 [0.21–1.53]	0.300

ECMO, extracorporeal membrane oxygenation; NIV, non-invasive ventilation; iMV, invasive mechanical ventilation; CRRT, continuous renal replacement therapy; ICU, intensive care unit; LOS, length of stay.

**Table 5:** Clinical condition before ECMO cannulation according to the hospital survival.

	Alive (n = 41)	Dead (n = 23)	OR [95% CI]	*p*-Value
Vasoactive drugs—n (%)	37 (90%)	18 (78%)	2.57 [0.63–9.05]	0.263
Pulmonary vasodilators—n (%)	9 (22%)	6 (26%)	0.80 [0.26–2.87]	0.766
NMBA—n (%)	39 (95%)	21 (91%)	1.86 [0.27–12.38]	0.614
Corticosteroids—n (%)	19 (46%)	12 (52%)	0.79 [0.30–2.07]	0.795
Prone position—n (%)	28 (68%)	14 (61%)	1.39 [0.47–3.87]	0.591
Pneumothorax—n (%)	4 (10%)	6 (26%)	0.31 [0.09–1.12]	0.148
Pneumomediastinum—n (%)	3 (7%)	4 (17%)	0.38 [0.09–1.54]	0.240
Subcutaneous emphysema—n (%)	3 (7%)	5 (22%)	0.28 [0.07–1.30]	0.124
Pleural effusion—n (%)	14 (34%)	8 (35%)	0.97 [0.35–2.81]	0.999
Pulmonary embolism—n (%)	3 (7%)	2 (9%)	0.83 [0.16–4.97]	0.999
Limb ischemia—n (%)	3 (7%)	0 (0%)	n.a.	0.547
Deep vein thrombosis—n (%)	5 (12%)	6 (26%)	0.39 [0.11–1.39]	0.182
NIV—n (%)	23 (56%)	13 (57%)	0.98 [0.36–2.58]	0.999
NIV (days)	1 [0; 2]	1 [0; 4]		0.358
iMV—n (%)	41 (100%)	23 (100%)	n.a.	0.999
iMV (days)	2 [1; 3]	2 [2; 3]		0.219
ICU LOS (days)	3 [1; 4]	2 [2; 4]		0.655
Hospital LOS (days)	3 [1; 5]	5 [2; 7]		0.051

ECMO, extracorporeal membrane oxygenation; NMBA, neuromuscular blocking agents; NIV, non-invasive ventilation; iMV, invasive mechanical ventilation; ICU, intensive care unit; LOS, length of stay; n.a., not applicable.

**Table 6:** Complications newly occurred after ECMO cannulation according to the hospital survival.

	Alive (n = 41)	Dead (n = 23)	IRR [95% CI]	*p*-Value
Patients with new complications—n/1000 days	9.8	63.5	0.15 [0.06–0.38]	<0.001
Pneumothorax—n/1000 days	8.9	37.0	0.24 [0.08–0.74]	0.008
Pneumomediastinum—n/1000 days	5.3	21.2	0.25 [0.06–1.21]	0.052
Subcutaneous emphysema—n/1000 days	5.3	26.5	0.20 [0.05–0.84]	0.016
Pulmonary embolism—n/1000 days	1.8	21.2	0.08 [0.01–0.59]	0.005
Pleural effusion—n/1000 days	2.7	37.0	0.07 [0.01–0.32]	<0.001
Hemothorax—n/1000 days	1.8	5.3	0.34 [0.02–19.8]	0.429
Acute myocardial infarction—n/1000 days	0.0	10.6	0.00 [0.00–0.89]	<0.001
Hemorrhagic brain injury—n/1000 days	0.0	5.3	0.00 [0.00–6.55]	0.015
Deep vein thrombosis—n/1000 days	0.9	21.2	0.04 [0.00–0.42]	0.002

IRR, incidence rate ratio; ECMO, extracorporeal membrane oxygenation; n.a., not applicable.

**Table 7:** Clinical outcomes according to hospital survival.

	Alive (n = 41)	Dead (n = 23)	OR [95% CI]	*p*-Value
Prone position during ECMO—n (%)	17 (42%)	15 (65%)	0.38 [0.14–1.14]	0.118
ECMO duration (days)	10 [8; 12]	4 [3; 5]		<0.001
NIV after ECMO cannulation—n (%)	30 (73%)	0 (0%)	n.a.	<0.001
iMV after ECMO cannulation (days)	14 [13; 18]	4 [3; 5]		<0.001
Total time spent under ventilatory support (days)	20 [19; 22]	7 [6; 11]		<0.001
Tracheostomy—n (%)	11 (27%)	2 (9%)	3.85 [0.89–18.53]	0.111
CRRT after ECMO cannulation—n (%)	15 (37%)	11 (48%)	0.63 [0.24–1.69]	0.433
Total ICU LOS (days)	27 [23; 32]	7 [6; 11]		<0.001
Total hospital LOS (days)	38 [33; 45]	10 [7; 12]		<0.001

ECMO, extracorporeal membrane oxygenation; NIV, non-invasive ventilation; iMV, invasive mechanical ventilation; CRRT, continuous renal replacement therapy; ICU, intensive care unit; LOS, length of stay.

## Data Availability

Data are available upon reasonable and scientific request from the corresponding author.
